# Nutritional evaluation and transcriptome analyses of short-time germinated seeds in soybean (*Glycine max* L. Merri.)

**DOI:** 10.1038/s41598-021-02132-2

**Published:** 2021-11-22

**Authors:** Wei Hu, Xiaoxue Liu, Yajun Xiong, Tingxuan Liu, Zhan Li, Jian Song, Jun Wang, Xianzhi Wang, Xiaofang Li

**Affiliations:** 1 College of Agriculture, Yangtze University, Jingzhou, 434025 Hubei People’s Republic of China; 2 College of Life Sciences, Yangtze University, Jingzhou, 434025 Hubei People’s Republic of China; 3 School of Agriculture, Yunnan University, Kunming, 650504 Yunnan People’s Republic of China

**Keywords:** Molecular biology, Plant sciences

## Abstract

Germination is a common practice for nutrition improvement in many crops. In soybean, the nutrient value and genome-wide gene expression pattern of whole seeds germinated for short-time has not been fully investigated. In this study, protein content (PC), water soluble protein content (WSPC), isoflavone compositions were evaluated at 0 and 36 h after germination (HAG), respectively. The results showed that at 36HAG, PC was slightly decreased (*P* > 0.05) in ZD41, J58 and JHD, WSPC and free isoflavone (aglycones: daidzein, genistein, and glycitein) were significantly increased *(P* < 0.05), while total isoflavone content was unchanged. Transcriptomic analysis identified 5240, 6840 and 15,766 DEGs in different time point comparisons, respectively. GO and KEGG analysis showed that photosynthesis process was significantly activated from 18HAG, and alternative splicing might play an important role during germination in a complex manner. Response to hydrogen peroxide (H_2_O_2_) was found to be down regulated significantly from 18 to 36HAG, suggesting that H_2_O_2_ might play an important role in germination. Expression pattern analysis showed the synthesis of storage proteins was slowing down, while the genes coding for protein degradation (peptidase and protease) were up regulated as time went by during germination. For genes involved in isoflavone metabolism pathway, *UGT* (7-O-glucosyltransferase) coding genes were significantly up regulated (40 up-DEGs vs 27 down-DEGs), while *MAT* (7-O-glucoside-6′′-O-malonyltransferase) coding genes were down regulated, which might explain the increase of aglycones after germination. This study provided a universal transcriptomic atlas for whole soybean seeds germination in terms of nutrition and gene regulation mechanism.

## Introduction

Soybean meal is one of the most important and preferred protein feed sources for poultry due to the capability of providing up to 40% protein and 20% oil, as well as its high nutritional value for suitable amino acid profile^1^. In developing and developed countries, foods are regarded as no more a source to only alleviate hunger, but provide the necessary nutrients for health care as well. How to enhance nutritional value of traditional staple foods has become a trend in modern food industry^2^.

Among different processing practices aiming at seed nutrition enhancement, germination is a common practice for nutrition improvement in many crops and is catching more and more special attention because the nutritional compositions are altered and new active substances are generated during the process. In cereals like rice and maize, germination is widely used to meliorate its nutritional value^3–6^. It is reported that, germinated brown rice has potential to create the highest value from rice by preserving all nutrients in the rice grain for human consumption^3^. By using combined processes of fermentation and germination in maize, protein and vitamin E content, total phenolic content, vitamin B1, and gamma-aminobutyric acid (GABA) content could be increased up to two, three, four, and five-fold respectively, and all these compounds were believed to be essential nutrients and played significant role for human health in terms of antioxidant activities^6^.

Generally, soybeans served as staple food in Asia, and as fodder for livestock and aquaculture as well^7^. Soybean foods derived directly from seeds, including soy flour, soy milk, soybean curd (tofu), yuba, textured protein et al.^8^, mainly make use of the major nutrients (i.e. protein and oil). While natto, miso, temph, sufu (*dou-fu-ru*), dajiang, and douchi et al., are fermented soybean foods with the purpose to improve its flavor and nutrition^9–12^. Besides, soybean sprouts derived from the late phase of germination is believed to be a year around vegetable with high nutrient^13–15^. Germination in soybean has been extensively studied as well. Basically, germination in soybean resulted in the reduction of anti-nutritional factors (Trypsin inhibitor activity)^16,17^, oil content, acid value, iodine value and total unsaturated fatty acids^18^, and the increasement of protein dispersibility^19^, protein solubility, total saturated fatty acids^18^, isoflavone content^8,13,20–23^, and other nutritional components.

Light is an important factor which trigger plant photomorphogenesis. The quality, intensity, and duration of light are believed to affect the seed germination and the secondary metabolism^24^. Studies showed that it was feasible to produce soybean sprouts enriched in isoflavones under colored-light sources^10^, and the application of combined light treatment of greenhouse lamps and ultraviolet light on 7-day-old Aga3 sprouts would result in maximum isoflavone production^25^. Research from Chickpea also suggested that the application of light is the most effective method for producing higher isoflavone contents in functional foods^26^. Although the light treatments in these studies was slightly different, the results inspired us to know how the light would impact the nutritional value on soybean seeds during early germination process.

In germination practice, the radicle length reaches around 5 cm after two days of germination, at which time the nutritional value of soybean seeds (e.g. isoflavone content) is still below its maximum (3–5 days)^27^. However, the milling capacity of germinated seeds drops with the germination time increases^19^, which is unfavorable if these germinated seeds are utilized for soy milk, bean curd, soy flour etc. The “trade-off” between milling capacity and nutritional improvement impels people to turn their attention to the early germination phase, expecting that soybean seeds germinated at early phase are still nutritional-improved. It is noticeable that there are plenty of soybean products utilizing germinated seeds as soymilk and rice supplement, e.g. Peimengdou and premium germinated black soybeans in China, which are very popular in health-conscious people.

During seed germination, seed storage proteins is mobilized to provide essential energy and carbohydrates for seedling establishment^28^. This process consists of the cleavage and breakdown of storage proteins catalyzed by proteolytic enzymes, such as peptidases (e.g. cysteine endopeptiase, serine endopeptidases, serine carboxypeptidase, aspartic endopeptidases, aminopeptidases, cytosolic dipeptidases), protease (aspartc proteinase, protease C1, protease C2, proteinase B, protease F), and hydrolase as well^28–30^. In soybean, germination could digest soybean proteins into smaller molecules, enhance the degree of hydrolysis, emulsifiability, and foaming capacity^27^. As phenolic nutrients, isoflavone have been reported to be implicated with the potentiality to reduce the risk of cancer, menopausal symptoms, cardiovascular disease and other kinds of age-related and hormone-related disorders^31^. Typically, there are four different forms of isoflavones, namely aglycones (daidzein, genistein, and glycitein), glucosides (daidzin, genistin, and glycitin), acetyl-glucosides (acetyldaidzin, acetylgenistin, and acetylglycitin), and malonyl-glucosides (malonyldaidzin, malonylgenistin, and malonylglycitin)^10,32^, of which, aglycones are free isoflavones, and the rest three forms are bounded isoflavones. Aglycones are bioactive forms of isoflavone, which could be absorbed faster and in higher amount than glucosides in humans^33^. In soybean, isoflavone metabolism is a branch of phenylpropanoid pathway. It starts with the conversion of phenylalanine into chalcone catalyzed by phenyl-alanine ammonia lyase (PAL; EC 4.3.1.5), cinnamate 4-hydroxylase (C4H; EC 1.14.13.11), 4-coumarate: coenzyme A ligase (4CL; EC 6.2.1.12), and chalcone synthase (CHS; EC 2.3.1.74)^34–36^. Chalcone were then transformed into isoflavanone (isoliquiritigenin and naringenin) by chalcone reductase (CHR; EC 2.3.1.170) and chalcone isomerase (CHI; EC 5.5.1.6) respectively^37,38^, which are committed step in isoflavone biosynthesis. Liquiritigenin, as the direct substrates for glycitein and daidzein, is isomerized from isoliquiritigenin by CHI. It is then converted into glycitein and daidzein by isoflavone synthase (IFS; EC 1.14.13.136) and 2-hydroxyisoflavanone dehydratase (2HID, EC 4.2.1.105)^39,40^. Daidzein could be further converted into glyceollin by several steps, including synthesis of glycinol by isoflavone reductase (IFR), isoflavone 2′-hydroxylase (I2′H), and pterocarpan 6α-hydroxylase (P6αH), and then from glycinol to glyceollin by glycinol 4-dimethylallyl transferase (G4DT), glycinol 2-dimethylallyl transferase (G2DT), and glyceollin synthase (GLS)^41,42^. For another isoflavone form, genistein is synthesized similar to glycitein and daidzein by IFS and 2HID, but from naringenin. Naringenin could not only be converted into isoflavone, but also a substrate for flavone and anthocyanins, which are catalyzed by flavone synthase (FNS; EC: 1.14.11.2) and flavanone 3-hydroxylase (F3H; EC 1.14.11.9), dihydroflavonol reductase (DFR; EC 1.1.1.219)^43–45^. Glucosides of acetyl-glucosides and malonyl-glucosides isoflavones are added by 7-O-glucosyltransferase (IF7GT/UGT; EC 2.4.1.170) and 7-O-glucoside-6′′-O-malonyltransferase (MAT; EC 2.3.1.115) respectively^46^. Although the isoflavone pathway in soybean has been extensively studied, how these genes are expressed during soybean seed germination remains unclear.

Although higher nutritional value has been suggested in hypocotyl and root part of soybean seeds^23,47^, and large-scale transcriptional analysis has been conducted on axis at early germination phase^48^, the nutrient value and genome-wide gene expression pattern of whole seeds germinated for short-time has not been fully investigated. Considering the importance of entire seeds, especially the cotyledon accounting for the most part of soybean seeds, the objectives of this study were to evaluate the major nutrients in whole seeds germinated for 36 h, and investigate the gene expression pattern dynamically at 0HAG (Hours After Germination), 18HAG, and 36HAG respectively. The results might provide theoretical basis for short-time germination of soybean in food industry.

## Results

### Major nutrients evaluation of germinated soybean seeds

**Figure 1:**
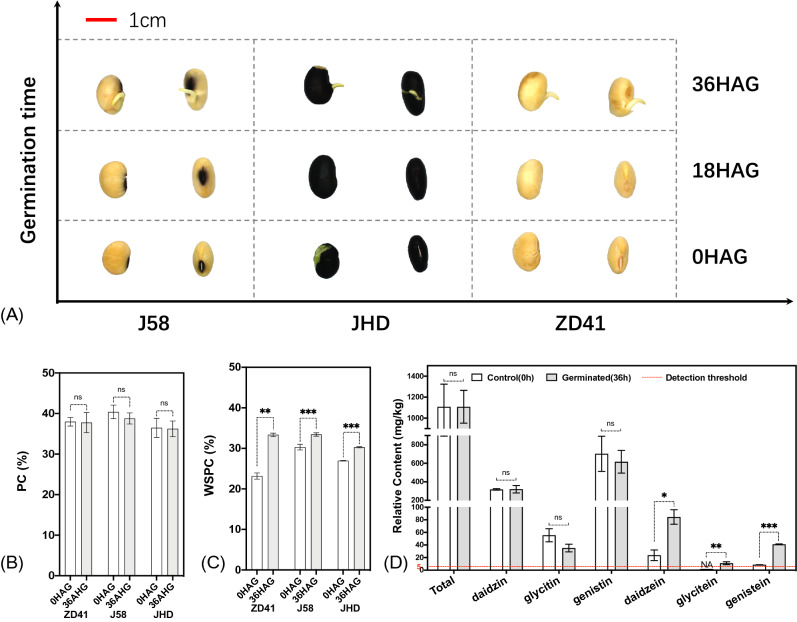
Nutrient alteration in germinated soybean seeds. (**A**) Representative seeds germinated for different time (0 h, 18 h, 36 h); (**B**, **C**) Protein content (PC) and water soluble protein content (WSPC) in germinated seeds, three independent replicates were performed; (**D**): isoflavone (daidzin, glycitin, genistin, daidzein, glycitein, and genistein) content in germinated seeds of three independent cultivars (ZD41, JHD, and J58) are compared between control (0 h) and germinated for 36 h. *, **, ***, indicates Student’s *t*-test significant *p* value of 0.05, 0.01, and 0.001 respectively, ns denotes non-significant.

In this study, three different cultivars with different seed coat and cotyledon color were employed to evaluate the impact of seed germination on major nutrient (i.e. protein, water soluble protein and isoflavone) in soybean. No rupture of seed coat was observed after germination for 18 h, and significant root growth was observed with the radicle length of 0.5–0.8 cm for different cultivars after germination for 36 h (Fig. 1A). Protein content (PC) decreased by 0.56%, 4.02%, and 0.70% for ZD41, J58 and JHD, respectively, but not significant (Fig. 1B). All three cultivars (ZD41, J58 and JHD) showed significant elevation of water soluble protein content (WSPC) at 36HAG in comparison with control (0HAG), of which ZD41 showed 30.52% increase, while J58 and JHD increased by 9.34% and 10.97%, respectively (Fig. 1C, *P* < 0.01). No significant change was observed for total isoflavone and glucosides-conjugated isoflavones in different cultivars (Fig. 1D). For glucosides-conjugated isoflavones in germinated soybeans, genistin showed the highest content with an average of 703.4 mg/kg, followed by daidzin with an average of 317.9 mg/kg and glycitin with an average of 55.5 mg/kg. However, of the free isoflavones (daidzein, glycitein, and genistein) in non-germinated soybean seeds, daidzein showed the highest content with an average of 23.6 mg/kg, followed by genistein with an average of 8.7 mg/kg, and glycitein was not detected in all three different cultivars. All these three free isoflavones significantly increased after germinated for 36 h but with varied extent (Fig. 1D, *P* < 0.05).

### Transcriptomic analysis during germination

To investigate the gene expression pattern of soybean seed during early germination, RNA-seq of whole seeds geminated for 0 h, 18 h, and 36 h were performed. Removing adaptive sequence and short reads, a total of 38.29 Gb data was obtained with an average of 5.97 Gb for each sample (Table 1). Generally, the base percent of Q30 (quality value larger than 99.9%) was > 94.55%, and over 90.01% of reads were mapped to soybean genome uniquely (Table 1), indicating that the sequencing quality was high.

**Table 1:** Statistic of RNA-seq data on the soybean genome.

Sample ID	Total reads	Mapped reads	Unique mapped reads	Multiple map reads	% ≥ Q30
0HAG.rep1	42,852,570	40,392,522 (94.26%)	39,038,995 (91.10%)	1,353,527 (3.16%)	94.69%
0HAG.rep3	42,281,130	39,692,718 (93.88%)	38,344,895 (90.69%)	1,347,823 (3.19%)	95.01%
18HAG.rep1	42,142,448	39,505,260 (93.74%)	38,190,551 (90.62%)	1,314,709 (3.12%)	94.74%
18HAG.rep3	46,109,094	43,153,740 (93.59%)	41,503,017 (90.01%)	1,650,723 (3.58%)	94.55%
36HAG.rep1	39,967,002	37,927,362 (94.90%)	36,828,432 (92.15%)	1,098,930 (2.75%)	95.15%
36HAG.rep2	42,946,438	40,626,235 (94.60%)	39,422,492 (91.79%)	1,203,743 (2.80%)	94.59%

**Figure 2:**
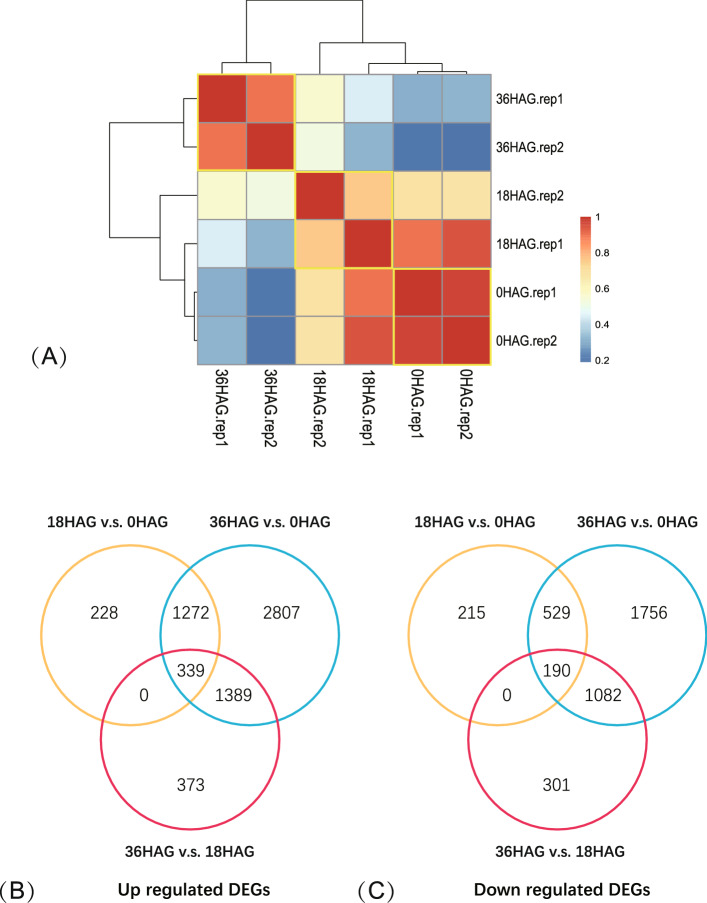
Pearson correlation coefficients between samples and DEGs identification. (**A**) Clustering of different samples based on Pearson correlation coefficients; (**B**, **C**) Venn map of up and down regulated DEGs respectively identified among different comparisons.

To evaluate the reliability of different biological replicates, correlation analysis was performed. The Pearson correlation coefficients between two replicates for 18HAG, 0HAG and 36HAG was 0.76, 0.97 and 0.90, respectively (Fig. 2A), indicating the high reliability of the RNA-seq data. Hierarchy clustering analysis showed that 18HAG and 0HAG were closely grouped together, indicating that little change was observed at 18HAG in comparison with non-germinated control, and vigorous change was occurred at 36HAG.

### DEGs identification during germination

Based on FPKM, different expressed genes (DEGs) were analyzed between different germination time points, i.e. 18HAG v.s. 0HAG, 36HAG v.s. 0HAG, and 36HAG v.s. 18HAG at |log_2_FC|≥ 2 (FC, Fold Change) threshold with false discovery rate ≤ 0.01. A total of 5240 DEGs were identified in comparison of 18HAG v.s. 0HAG, of which 2744 DEGs were up regulated and 2496 were down regulated (Fig. 2B, C, Table S1). In the 36HAG v.s. 0HAG comparison, 6840 DEGs were identified, of which 3809 and 3031 DEGs were up and down regulated, respectively (Fig. 2B, C, Table S1). Notably, a total of 15,766 genes were differentially expressed after germinated for 36 h compared with 0 h, of which 9144 and 6612 DEGs were up and down regulated, respectively (Fig. 2B, C, Table S1). The number of DEGs increased along with the germination time, and more up regulated DEGs were observed than down regulated DEGs during the germination process, indicating that metabolism activation was dominant during early stages of seed germination in soybean.

### GO analysis of DEGs

**Figure 3:**
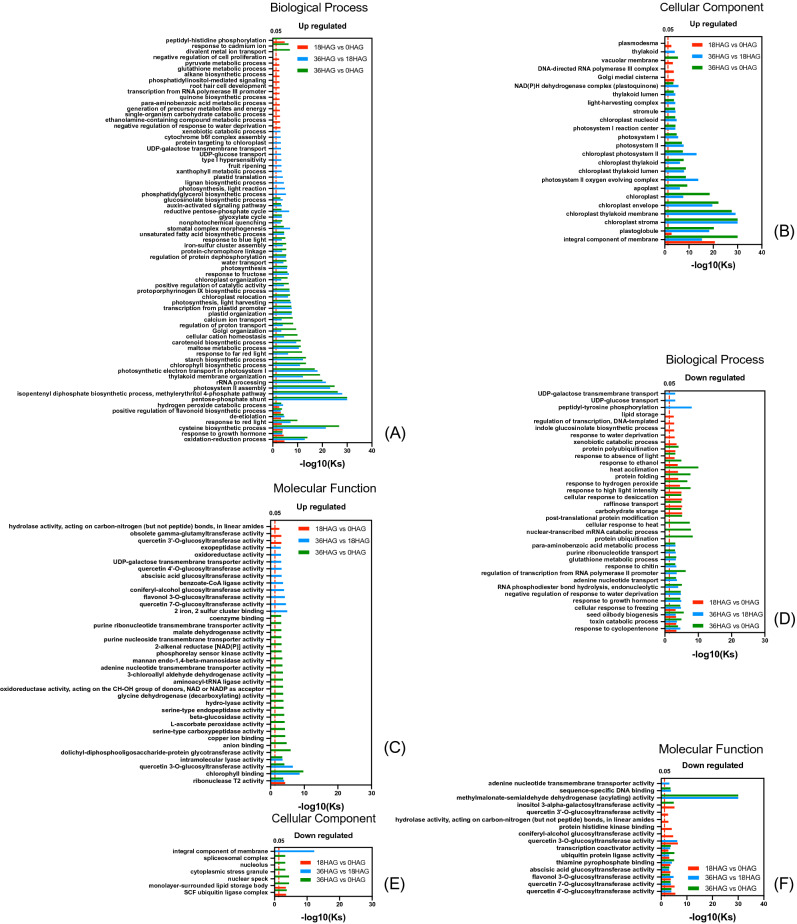
GO enrichment of DEGs during short-time germination. Biological process (**A**), cellular component (**B**), molecular function (**C**) of up regulated DEGs, Biological process (**D**), cellular component (**E**), molecular function (**F**) of down regulated DEGs.

In this study, transcriptomic analysis demonstrated that more genes were up regulated than down regulated (15,697 vs. 12,139) during short-time germination process (0HAG, 18HAG and 36HAG, Fig. 2, Table S1), suggested a clear activation of metabolism at early phase of germination. In order to better understand what these DEGs are and how they are involved in the germination process, GO (Gene Ontology) analysis were performed for comparison between each germination time point. GO enrichment analysis could provide us essential information about which biological process, cellular component, and molecular function are significantly associated with DEGs. At the very beginning of germination (0HAG-18HAG), seeds absorbed water from environment, resulted in negative response to water deprivation before 18HAG. During the same time, precursor metabolites and energy was activated, metabolic process of intermediate compounds (para-aminobenzoic acid, single organism carbohydrate, ethanolamine-containing compounds, glutathione, and pyruvate) was initiated. Meanwhile, root hair cells were up regulated and enriched (Fig. 3A, Table S2). After that, a highlighted change was observed for the mobilization of photosynthesis related processes, including genes enriched in chlorophyll biosynthetic process, protein targeting to chloroplast, thylakoid membrane organization, protoporphyrinogen IX biosynthetic process, chloroplast relocation, chloroplast organization, photosynthetic electron transport in photosystem I, photosystem II assembly, light harvesting and reaction (response to far red and blue light), photosynthesis (Fig. 3A, Table S2). Along with the photosynthesis mobilization, carbon metabolism was activated as well (Fig. 3A, Table S2), including starch biosynthetic process, pentose-phosphate shunt, maltose metabolic process, glyoxylate cycle, reductive pentose-phosphate cycle, UDP-glucose transport, UDP-galactose transmembrane transport, isopentenyl diphosphate biosynthetic process, methylerythritol 4-phosphate pathway, response to fructose. Other up-regulated biological processes were also observed, including fatty acid metabolism (unsaturated fatty acid biosynthetic process, phosphatidylglycerol biosynthetic process), secondary metabolism (carotenoid biosynthetic process, glucosinolate biosynthetic process, lignan biosynthetic process), ion transport and homeostasis (cellular cation homeostasis, regulation of proton transport, calcium ion transport), plant growth regulation (positive regulation of catalytic activity, regulation of protein dephosphorylation, auxin-activated signaling pathway), plastid protein synthesis (rRNA processing, transcription from plastid promoter, plastid translation), and water transport. Notably, oxidation–reduction, response to growth hormone, cysteine biosynthesis, response to red light, de-etiolation, positive regulation of flavonoid biosynthesis, and hydrogen peroxide catabolism were up regulated since 0HAG, and lasted to 36HAG, suggesting that these biological processes were sensitive to germination and might play important roles in seedling morphogenesis. In terms of molecular function, these up regulated DEGs were mainly enriched in chlorophyll binding and quercetin 3-O-glucosyltransferase (Fig. 3C, Table S2), which were involved in photosynthesis and isoflavone synthesis, respectively.

Cellular localization analysis of proteins encoded by the up regulated genes showed that most of them enriched in integral component of membrane, plastoglobule, Golgi medial cisterna, DNA-directed RNA polymerase III complex, plasmodesma, and vacuolar membrane at the very beginning (18HAG v.s. 0HAG). After that, most of them tended to located in chloroplast (e.g. chloroplast, chloroplast stroma, thylakoid membrane, thylakoid lumen, chloroplast envelope, chloroplast photosystem II, photosystem II oxygen evolving complex, photosystem I, photosystem I reaction center, chloroplast nucleoid, light-harvesting complex, NAD(P)H dehydrogenase complex), apoplast, and stromule (Fig. 3B, Table S2).

Down regulated DEGs related to xenobiotic catabolic process, lipid storage, response to water deprivation, indole glucosinolate biosynthetic process, and regulation of transcription and DNA-templated were repressed at the very beginning (from 0 to 18HAG) (Fig. 3D, Table S2). After that, stress response (negative regulation of response to water deprivation, response to chitin, glutathione metabolic process), hormone crosstalk and response (response to growth hormone, para-aminobenzoic acid metabolic process), nucleotide acid transport, regulation and degradation (RNA phosphodiester bond hydrolysis, adenine nucleotide transport, purine ribonucleotide transport, regulation of transcription from RNA polymerase II promoter) were reduced from 18 to 36HAG (Fig. 3D, Table S2). Furthermore, stress response (response to cyclopentenone, toxin catabolic process, cellular response to freezing, cellular response to desiccation, response to hydrogen peroxide, response to ethanol, heat acclimation), seed oilbody biogenesis, carbohydrate anabolism (carbohydrate storage, raffinose transport), light intensity response (response to high light intensity, response to absence of light), protein synthesis and degradation (protein folding, protein polyubiquitination) kept inhibited during the whole early phase of germination (Fig. 3D, Table S2). For the molecular function of these down regulated DEGs, methylmalonate-semialdehyde dehydrogenase (acylating) activity was mostly enriched (Fig. 3F, Table S2). Compared with the up regulated DEGs, down regulated DEGs were much widely distributed in SCF ubiquitin ligase complex, monolayer-surrounded lipid storage body, nuclear speck, cytoplasmic stress granule, nucleolus, spliceosomal complex, and integral component of membrane.

### KEGG Clustering of gene expression profile during germination

In this study, GO analysis of DEGs of either up or down regulated were performed, which provided us essential information of biological process, molecular function and cellular components involved, however not relevant to specific pathway. To compensate the disadvantage, hierarchy clustering of overall DEGs and KEGG pathway enrichment were then conducted with the expectation to gain an overall view of expression pattern of all DEGs.

**Figure 4:**
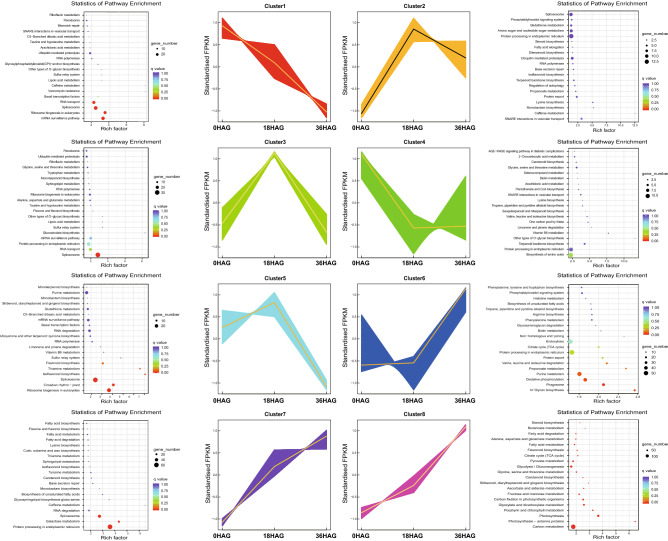
Hierarchy clustering of overall gene expression. Gene expression pattern of all DEGs are classified into 8 clusters, which is displayed by line chart within middle of this figure. KEGG enrichment of DEGs within each cluster are displayed by bubble chart alongside each line chart.

Hierarchy clustering of overall gene expression showed that 18HAG was grouped with 0HAG, indicating that gene expression change was occurred mainly after 18 h of germination. Specifically, DEGs grouped into eight clusters. Based on the expression pattern of 18HAG in comparison to 0HAG, these clusters could be classified into three groups, namely I (cluster 1 and 8), II (cluster 2, 5, and 6), and III (cluster 3, 4, and 7) (Fig. 4, Tables S3, S4). In group I, expression level of genes at 18HAG showed similar expression as that of 0HAG, and for 36HAG genes were dramatically up regulated in cluster 1 (1757 genes), but down regulated in cluster 8 (5536 genes). The genes within cluster 1 were enriched in four KEGG pathways, namely mRNA surveillance pathway, ribosome biogenesis in eukaryotes, RNA transport, and spliceosome. While in cluster 8, most genes were enriched in at least 20 pathways, most of which were relevant to carbon metabolism, photosynthesis-antenna proteins, and photosynthesis. For clusters within group II, expression level of genes from all three clusters were dramatically up regulated at 18HAG compared with that of 0HAG. But for 36HAG, expression level of genes was stable in cluster 2, decreased in cluster 5, and increased in cluster 6 (Fig. 4, Tables S3, S4). KEGG analysis showed that genes from cluster 2 enriched in none pathways, genes from cluster 5 enriched in ribosome biogenesis in eukaryotes, circadian rhythm-plant, spliceosome, isoflavonoid biosynthesis, thiamine metabolism and flavonoid biosynthesis, and genes from cluster 6 were mostly enriched in N-glycan biosynthesis, phagosome, oxidative phosphorylation, purine metabolism, propanoate metabolism, and amino acid (valine, leucine, and isoleucine) degradation. For group III, gene expression level of 18HAG all showed decreased trend compared with that of 0HAG, but for 36HAG, expression level of genes kept stable in cluster 7, decreased in cluster 3, and increased in cluster 4 (Fig. 4, Tables S3, S4). For cluster 3, spliceosome was found to be enriched and down regulated continuously after germination. For cluster 4, there were no significant pathway enriched. For cluster 7, protein processing in endoplasmic reticulum, galactose metabolism, and spliceosome were enriched. Interestingly, of these pathways enriched in cluster 1, 3, 5, and 7, spliceosome was consistently identified, indicating that the spliceosome pathway was down regulated at 36HAG but varied at 18HAG (Fig. 4, Tables S3, S4).

### Expression pattern analysis of genes involved in protein mobilization

**Figure 5:**
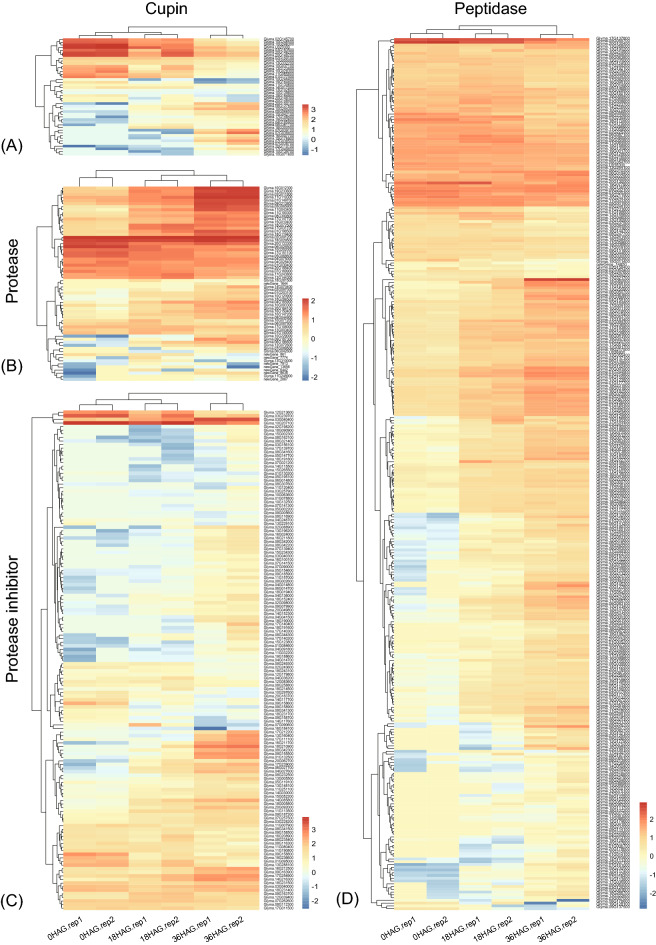
Heatmap of expression pattern of genes involved in protein metabolism. (**A–D**) expression pattern of genes coding for cupin, protease, protease inhibitor, and peptidase respectively. FPKM of each gene was transformed by log10 before heatmaping.

Significant increasing of WSPC was observed at 36HAG in all three cultivars in this study. To better understand the mechanism behind, genes coding for cupin, protease, protease inhibitor and peptidase were studied. A total of 44 cupin (or cupin domain containing) coding genes were identified to be changed at transcriptional level during germination. Of which, 10 genes annotated to be coding for glycinin (G1, G3, G4, G7, A4B4) and conglycinin (alpha, alpha prime, beta), which accounts for the majority of soybean storage protein, were all down regulated either at 18HAG or 36HAG in comparison with 0HAG (Table S5; Fig. 5A). Other down-regulated genes including genes coding for vicilin-like protein, pirin-like protein, sucrose binding protein (Table S5). On the contrary, 14 up-regulated coding genes for cupin were identified, five of which were found to be coding for auxin-binding proteins ABP19a, and up regulated for at least eightfold (log2FC > 3) after germination. *Glyma.08G127600,* coding for 13S globulin-like protein, was up regulated to ~ 32 fold (log2FC > 5) at 36HAG compared with 0HAG.

A total of 316, 63, and 139 genes coding for peptidase, protease and protease inhibitor were identified, respectively (Figs. 5B, C, D; Table S5). In general, more up-regulated than down-regulated genes were observed in peptidase and protease when compared with 0HAG. For peptidase, 69 and 30 genes were up and down regulated at 18HAG compared with 0HAG, while 209 and 81 genes were up and down regulated at 36HAG compared with 0HAG (Fig. 5D, Table S5). Similar trend was observed in protease, in which 11 and 8 genes were up and down regulated at 18HAG, while 36 and 20 genes were up and down regulated at 36HAG compared with 0HAG (Fig. 5B, Table S5). However, more down-regulated than up-regulated genes were observed in protease inhibitor. At 18HAG compared with 0HAG,, 16 and 21 genes were up and down regulated, while 87 and 40 genes were up and down regulated at 36HAG in comparison with 0HAG (Fig. 5C, Table S5). These findings might provide overall information underlying the mechanism of improved protein water solubility.

### Isoflavone metabolism related gene expression analysis

**Figure 6:**
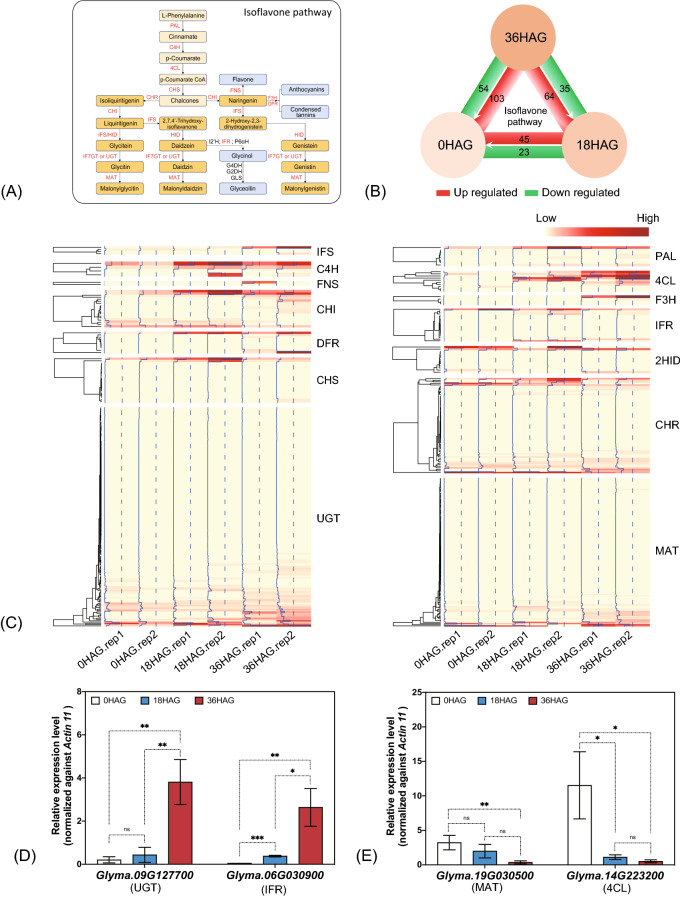
Expression pattern of genes involved in isoflavone metabolism. (**A**) Isoflavone pathway (details could be found in the Introduction section); (**B**) up and down regulated gene number between different germination time points (18HAG v.s. 0HAG, 36HAG v.s. 18HAG, 36HAG v.s. 0HAG); (**C**) Heatmap of coding genes expression pattern for IFS (isoflavone synthase), C4H (cinnamate 4-hydroxylase), FNS (flavone synthase), CHI (chalcone isomerase), DFR (dihydroflavonol 4-reductase), CHS (chalcone synthase), IF7GT/UGT (isoflavone 7-O-glucosyltransferase), PAL (phenylalanine ammonia-lyase), 4CL (4-coumarate-CoA ligase), F3H (flavanone 3-hydroxylase), 2HID (2-hydroxyisoflavanone dehydratase), CHR (chalcone reductase), MAT (isoflavone 7-O-glucoside 6"-O-malonyltransferase), IFR (isoflavone reductase); (**D**, **E**) qRT-PCR confirmation of representative genes UGT: *Glyma.09G127700*; IFR: *Glyma.06G030900*; MAT: *Glyma.19G030500*; 4CL: *Glyma.14G223200*) in isoflavone metabolic pathway. Relative expression level of each gene was normalized against *Actin 11*. *, **, ** indicates significant level of *P* < 0.05, 0.01, and 0.001 respectively by Student’s *t*-test, ns denotes none significant. Dashed line represents several steps.

As described above, three free isoflavone contents in germinated seeds were significantly changed. To better understand the expression pattern of genes involved in isoflavone metabolism, the isoflavone metabolism pathway was summarized in Fig. 6A. In this study, a total of 483 genes belonging to 14 functional classes (responsible for different enzymatic steps) were identified based on historical literature and homologues annotated by Wm82 a2.v1 (Tables S6, S7). Of which, 108 genes (22.36% of total) were identified to be quiescent (undetectable) in different time points, and 299 genes (61.9%) showed unchanged during germination (Table S7). For different time point comparison, 45 and 23 genes were up and down regulated at 18HAG compared with 0HAG, 64 and 35 genes were up and down regulated at 36HAG in comparison with 18HAG, and 103 and 54 genes were up and down regulated at 36HAG in comparison with 0HAG, respectively (Fig. 6B). At the entry point of isoflavone metabolism, genes coding for PAL, C4H showed either up regulated or unchanged, and most genes coding for 4CL showed up or unchanged regulation pattern, except for *Glyma.14G223200* (Tables S7, S8). For CHI, CHS, and CHR, which are responsible for the synthesis of isoflavone precursors (isoliquiritigenin, naringenin), more genes were up regulated than down regulated. For IFS, only one coding gene was up regulated, and two genes were down regulated (Fig. 6B; Tables S7, S8). Besides converting into genistein, naringenin is the fork point for flavone and anthocyanins branches. Interestingly, FNS coding genes were undetectable in this study, while F3H and DFR coding genes were up regulated (Fig. 6B; Tables S7, S8). For genes responsible for glycosylation of isoflavone, more isoflavone 7-O-glucosyltransferase (IF7GT/UGT) coding genes were up regulated, (i.e., 40 and 27 genes were up and down regulated, respectively), and similar trend was observed in genes coding for isoflavone 7-O-glucoside 6"-O-malonyltransferase (MAT), of which, 21 and 15 genes were up and down regulated, respectively (Fig. 6C; Tables S7, S8). With regards to catabolism of daidzein, IFR were found to be up regulated (Fig. 6C; Tables S7, S8). To confirm the gene expression pattern identified by RNA-seq, several representative genes coding for important enzymes involved in isoflavone metabolism pathway, e.g. 4CL, UGT, IFR, and MAT, were quantified by qRT-PCR. Results showed that *Glyma.09G127700* (*UGT*), and *Glyma.06G030900* (*IFR*) were up regulated at 18HAG and 36HAG, and *Glyma.19G030500* (*MAT*) and *Glyma.14G223200* (*4CL*) were down regulated, which was consistent with RNA-seq results (Fig. 6D, E; Table S7).

## Discussion

### Nutritional value improvement in short-time germinated soybean seeds

In this study, the major nutrients in germinated soybean seeds were evaluated. The results showed that no significant increase of PC was observed for soybean seeds germinated for 36 h in all three cultivars (Fig. 1B, *P* > *0.05*), which was not consistent with previous studies^49,50^. This might because the soybean seeds were only soaked for 4 h and germinated for 36 h in this study, while soybean seeds were treated much longer in previously studies. However, WSPC were significantly increased by 30.52%, 9.34%, and 10.97% in ZD41, J58 and JHD, respectively (Fig. 1C *P* < *0.01*), which is consistent with previous studies^18^. This could be due to possible breakdown of soy proteins into smaller molecules and the consequently enhanced degree of hydrolysis^27^. Although no significant increase of crude protein content was observed in this study, the elevated solubility of protein should be beneficial for the nutrient improvement of short-time geminated soybean seeds.

In this study, the contents of aglycones and glucosides were evaluated as well. No significant change for total isoflavone content was observed after short-time germination (i.e. 36 h) in all three different cultivars (Fig. 1D), which contradicted the conclusion that germination can help to increase total isoflavone content^13^. This could be happened in several situations, e.g. the germination time was not enough, not all compositions of isoflavone were tested (e.g. acetyl-glucosides, and malonyl-glucosides), or varied genetic background, which needs further investigations.

In comparison with non-germinated soybean seeds, germinated seeds showed stable content of daidzin, but reduced contents of glycitin and genistin although not significantly different. On the contrary, the contents of all three free isoflavones (i.e. daidzein, glycitein, and genistein) were significantly increased after germination for 36 h in all three cultivars (Fig. 1D *p* < *0.05*), which confirmed the previous conclusion^13,51^. Taken together, the results could suggest that the elevation of free aglycones content is a universal phenomenon during germination in soybean.

### Gene expression pattern of entire soybean seeds at early phase of germination

It is noteworthy that a substantial amount of up-regulated DEGs were enriched in photosynthesis and located on chloroplast related cellular compartments (Fig. 3B, Table S2) after 18HAG, indicating that the light morphogenesis was initiated at the very beginning of germination under continuous illumination conditions.

Germination started with the imbibition. In our study, negative regulation of response to water deprivation was activated at 18HAG, and then was repressed at 36HAG compared with control (Fig. 3, Table S2). This might probably because that at the beginning seeds already absorbs enough water during imbibition, but when it comes to the 36HAG, seed germination requires more water than 18HAG and consequently resulted in the block down of negative regulation of water response. We also noticed that carbohydrate anabolism was repressed and metabolism was activated (confirmed by KEGG enrichment of cluster 8), which is consistent with previous studies^52^. Enzymes involved in unsaturated fatty acid biosynthesis was up regulated, which is contradicting with the previous results^18^. This is probably because continuous light was adopted in this study which was different from the previous studies.

Previous study revealed that continuous white light resulted in higher testa and endosperm rupture rates, and a complex network among abscisic acid (ABA), gibberellin acid (GA) and hydrogen peroxide (H_2_O_2_) signaling pathways worked when seeds germinated in darkness in *Arabidopsis*, within which H_2_O_2_ played an important role in downstream of cell wall loosening and a upstream signal governing the light-dependent germination process^53^. In this study, hydrogen peroxide catabolic process was up regulated and the biological process response to hydrogen peroxide was down regulated from 18 to 36HAG (Fig. 3A, D, Table S2). This is probably because radicle length of soybean seeds germinated for 36 h reached 0.5 cm, and the testa rupture process was already ended (Fig. 1A).

In this study, slightly decreased PC but significantly increased WSPC in germinated soybean seeds at 36HAG were observed, suggesting that protein solubility was improved by germination. Interestingly, a certain transcriptional level of genes for seed storage proteins was identified at 0HAG, and most of which were down regulated as germination time extended to 18 and 36HAG, except for the gene coding for 13S globulin-like protein. These results suggested that synthesis of seed storage proteins was slowing down during germination. On the other side, gene expression pattern analysis of peptidase and protease, which are responsible for the degradation of proteins, showed strikingly increased number of up-regulated genes as the germination time extended. This might explain the reason why germination will result in improved protein solubility (increased WSPC). However, increased gene number of protease inhibitor was observed in germinated seeds as well, implying that a complex “trade-off” regulation network might be existed in germinating seeds.

For genes coding for enzymes (PAL, C4H, and 4CL), which is located at entry point of phenylpropanoid pathway, showed up-regulated pattern as the time went by during germination, strongly suggesting an activation of phenylpropanoid pathway. After p-Coumarate CoA was synthesized, more *CHS*, *CHI*, and *CHR* genes was observed to be upregulated, indicating the possibility of accumulated chalcones, isoliquiritigenin, and naringenin. While in the branch towards flavone and anthocyanins, FNS was not activated, indicating that flavone synthesis was activated. However, significant increasement for the expression of *F3H* and *DFR* were observed, suggesting that anthocyanins or condensed tannins was probably increased. In this study, no significant change for total isoflavone content was observed, this is probably because that more *IFS* were down regulated than up regulated. Furthermore, more IF7GT/UGT coding genes were identified to be up regulated than down regulated, might implying that IF7GT/UGT responsible for the conversion between aglycones and glucosides might be activated. These finding is consistent with observation that aglycones were significantly increased in germinated seeds.

## Materials and methods

### Plant materials

In this study, Zhongdou 41 (ZD41, yellow seed coat and yellow cotyledon), Jingheidou (JHD, black seed coat and green cotyledon) and Jing58 (J58, yellow seed coat and yellow cotyledon) were utilized for germination and nutrient evaluation. The cultivars were selected based on their good performance with good adaptability and high yield in Jingzhou, Hubei Province. Zhongdou 41 is provided by Oil Crop Research Institute, Chinese Academy of Agricultural Sciences. JHD and J58 are two breeding lines obtained via personal communication. Three cultivars were planted in Field Test Center of Yangtze University in Jingzhou, Hubei Province, during the normal growing season in 2017. Seeds were harvested and stored under 4℃ to keep seed vigor before seed germination and nutritional evaluation.

### Seed germination and nutritional evaluation

Approximately 1.5 kg seeds of ZD41, J58 and JHD were sterilized and rinsed by deionized water for three times, then soaked in deionized water for 4 h for fully imbibition. After that seeds were placed on wetted gauze supported by plain stainless-steel tray under 28℃ (dark) in culture room with 80% humidity for 0 h, 18 h, and 36 h, respectively (hereafter designated as 0HAG, 18HAG and 36HAG respectively). Illumination condition was setup to 24 h continuous white light of 5500 lx as suggested^54^. Geminated seeds were inactivated at 95℃ for 30 min immediately, and then dried under 60℃ to constant weight. Protein content (PC), water soluble protein content (WSPC), were evaluated by Kjeldahl method^55^. Isoflavone composition was determined using HPLC as described previously^13^. Germinated seeds of three cultivars were performed for 3 biological replicates, and untreated seeds were used as control.

### RNA-seq

The seeds of ZD41 were germinated according to methods and protocols described above for two biological replicates. Whole seeds were frozen immediately after germination in liquid nitrogen and stored at -80℃ for further analysis.

Total RNA was extracted using TRIzol kit (Thermo Fisher Scientific, Cat No. 15596026) and quantified by Nanodrop 2000 (Thermo Fisher Scientific, USA) according to the users’ manual. Sequencing library were constructed by NEBNext® Ultra™ RNA Library Prep Kit for Illumina® (NEB, USA). Generally, mRNA of total RNA was purified by poly-T oligo-attached magnetic beads, and then fragmented using divalent cations under elevated temperature. First strand cDNA was then synthesized using random hexamer primer, M-MuLV Reverse Transcriptase (RNase H free) and DNA polymerase I, RNase H. After that overhangs remained were converted into blunt ends and the 3’ ends of DNA fragments was adenylated using exonuclease/polymerase and ligase. cDNA of 150-200 bp were enrich by High-Fidelity PCR to obtain final cDNA library. Finally, 100 bp paired-end reads were generated by Illumina Hiseq 2000 platform.

### GO and KEGG of differentially expressed genes (DEGs)

The clean reads were mapped to the Wm82 a2.v1 using Bowtie2^56^. The gene expression level of each gene was estimated by RSEM^57^ and normalized by the FPKM (fragments per transcript kilobase per million fragments mapped)^58^. Differentially expressed genes (DEGs) between different comparisons, namely 18HAG v.s. 0HAG, 36HAG v.s. 0HAG and 36HAG v.s. 18HAG, were defined as the fold change (FC) larger than 4 (|log_2_FC|≥ 2).

Gene Ontology (GO) enrichment analysis of the differentially expressed genes (DEGs) was implemented by the GOseq R packages based Wallenius non-central hyper-geometric distribution^59^, which can adjust for gene length bias in DEGs. KEGG analysis were performed by KOBAS 2.0^60,61^. Hierarchy clustering of overall DEGs were performed using MultiExperiment Viewer v4.7.4^62^.

### Gene expression pattern analysis

Protein metabolism related genes were filtrated from Table S1 using keywords of cupin, protease, protease inhibitor, and peptidase. Isoflavone metabolism pathway related genes were pyramided from literature (Table S6), combined with homologues of these gene identified based on functional annotation by Wm82 a2.v1 (www.phytozome.net). Heatmaps of FPKM of each genes were displayed using pheatmap packages in R v4.0^63^. Hierarchy clustering method were used for gene cluster analysis.

### qRT-PCR analysis

To performed qRT-PCR, seeds germinated at 0HAG, 18HAG, and 36HAG were stored in liquid nitrogen immediately after germination. qRT-PCR were performed basically according to Zhang et al.^64^ with modifications. Total RNA was isolated by TRNzol (cat. no. DP424; Tiangen Biotech Co., Ltd.), and the first strand of cDNA was synthesized using FastKing gDNA Dispelling RT SuperMix (cat No.KR118-02; Tiangen Biotech Co., Ltd.). Relative expression level of above mentioned four genes were calculated by the delta-delta-cycle threshold (C_t_) method^65^ using *Actin 11* as internal standard^66^. Gene specific primers for qRT-PCR were listed in Table S9. Student’s t-test was used to analyze qRT-PCR data statistically by GraphPad Prism v8^67^.

### Ethical approval

All the experiments carried out on plants in this study were in compliance with relevant institutional, national, and international guidelines and legislation.

## Supplementary Information

Supplementary Information 1.

Supplementary Information 2.

Supplementary Information 3.

Supplementary Information 4.

Supplementary Information 5.

Supplementary Information 6.

Supplementary Information 7.

Supplementary Information 8.

Supplementary Information 9.

## Data Availability

All data and material were available in the supplementary files.
